# *In vitro* inhibitory effects of gentamicin and ceftiofur against *Trypanosoma evansi*: Promising antibiotic alternatives for equine trypanosomosis in Thailand

**DOI:** 10.14202/vetworld.2025.3779-3787

**Published:** 2025-12-10

**Authors:** Apiraya Rudeekiatthamrong, Giang Thi Nguyen, Ketsarin Kamyingkird

**Affiliations:** Department of Parasitology, Faculty of Veterinary Medicine, Kasetsart University, Lad Yao, Chatuchak, Bangkok, Thailand.

**Keywords:** ceftiofur, diminazene aceturate, equine trypanosomosis, gentamicin, *in vitro* inhibition, *Trypanosoma evansi*

## Abstract

**Background and Aim:**

*Trypanosoma evansi* infection (Surra) remains a major constraint to equine health and productivity in Thailand. The only available trypanocidal drug, diminazene aceturate (DA), has limited efficacy, poor blood–brain barrier penetration, and toxicity in horses. This study aimed to investigate the *in vitro* inhibitory effects of commonly used equine antibiotics, gentamicin (GMC), ceftiofur (CTF), and trimethoprim–sulfamethoxazole (TS), against *T. evansi* (Thai strain isolated from dairy cattle number 953; TEDC 953) to identify potential therapeutic alternatives or adjuncts for equine trypanosomosis.

**Materials and Methods:**

An *in vitro* growth inhibition assay was conducted using the *T. evansi* TEDC 953 strain cultivated in Hirumi’s Modified Iscove’s medium 9 (HMI-9 medium) containing 20% horse serum under controlled conditions (37°C, 5% CO_2_, 75% humidity). Serial dilutions of DA, GMC, CTF, and TS were tested in duplicate across three independent experiments. Parasite viability was assessed after 48 h by microscopic examination, and the half-maximal effective concentration (EC_50_) was determined using nonlinear regression analysis in GraphPad Prism 5.

**Results:**

Among the three antibiotics, GMC and CTF significantly inhibited *T. evansi* growth *in vitro*, whereas TS showed no inhibitory effect. The EC_50_ values were 1.25 × 10^-5^ ± 3.90 × 10^-6^ mg/mL for DA, 0.22 ± 0.08 mg/mL for GMC, and 0.08 ± 0.05 mg/mL for CTF. Parasite viability assays confirmed that GMC (5 mg/mL) and CTF (0.2 mg/mL) completely eliminated *T. evansi* after 48 h of exposure. These findings provide the first *in vitro* evidence of the trypanocidal potential of GMC and CTF against the Thai strain of *T. evansi*.

**Conclusion:**

GMC and CTF exhibited substantial inhibitory activity against *T. evansi* under *in vitro* conditions, supporting their potential use as repurposed or adjunct antibiotics for trypanocidal therapy in horses. This preliminary evidence underscores the need for *in vivo* validation, pharmacokinetic profiling, and mechanistic studies to explore synergistic effects with conventional trypanocides such as DA.

## INTRODUCTION

*Trypanosoma evansi* is a flagellated protozoan parasite primarily transmitted by hematophagous insects. Among all trypanosome species, *T. evansi* has the broadest host range and worldwide distribution [1], with horses among the most severely affected hosts [2]. Clinical manifestations in infected horses include high fever, anemia, lethargy, weakness, lymphadenopathy, and neurological disturbances [2]. In acute cases, mortality rates can exceed 50%, and death may occur within three months of infection [3]. In Thailand, *T. evansi* outbreaks are reported annually, resulting in substantial economic losses to the horse-breeding industry [4].

Currently, there is no vaccine available for *T. evansi*, and treatment relies solely on trypanocidal drugs such as polysulfonate naphthylamine, quinapyramine sulfate and chloride, melarsomine, homidium salts, and diminazene aceturate (DA) [4, 5]. However, these drugs are often expensive, difficult to access, and unavailable in remote regions, prompting livestock owners to explore alternative or adjunct therapies. In some cases, trypanocidal drugs have been combined with antibiotics or ethnoveterinary (herbal) remedies to treat trypanosomosis, particularly in camels [6]. Infection in horses not only compromises animal health and performance but also affects international animal movement and trade. A major limitation remains the scarcity of effective compounds capable of completely eliminating *T. evansi* infection in equines.

Veterinary antibiotics commonly used in horses may offer a promising alternative or supportive role in trypanosomosis treatment because of their safety and widespread availability. These include eleven major classes: β-lactams, aminoglycosides, sulfonamides, tetracyclines, macrolides, ansamycins, fluoroquinolones, nitroimi-dazoles, phenicols, polypeptides, and streptogramins [7–9]. Gentamicin (GMC), an aminoglycoside antibiotic, is widely used against Gram-negative bacterial infections. It inhibits protein synthesis by binding to the A-site of 16S ribosomal RNA on the 30S ribosomal subunit with high affinity [10]. GMC is considered safe for intramuscular and intravenous administration in both adult horses and foals [8]. Experimentally, GMC has shown efficacy in treating *T. evansi* infection in goats [11] and has been reported to successfully treat a *T. lewisi*-like infection in an infant [12].

Ceftiofur (CTF), a third-generation cephalosporin belonging to the β-lactam class [8, 9, 13], exhibits broad activity against both Gram-positive and Gram-negative bacteria. β-lactams act by disrupting bacterial cell wall synthesis by inhibiting penicillin-binding proteins, although they do not cross the blood–brain barrier [14, 15]. CTF is widely used in horses and foals due to its safety and efficacy [8]. A previous study by Singla *et al*. [16] suggests that CTF may enhance DA penetration into erythrocytes, thereby improving DA pharmacokinetics and therapeutic outcomes in trypanosomosis.

Trimethoprim–sulfamethoxazole (TS), a combination of two sulfonamide derivatives [17], is an effective broad-spectrum antimicrobial for equine bacterial infections [8]. However, it is unsuitable for treating infections involving purulent or necrotic tissue [18]. Trimethoprim inhibits dihydrofolate reduction to tetrahydrofolate, while sulfonamides inhibit folate synthesis by competing with para-aminobenzoic acid [18, 19]. TS can be safely administered orally or intravenously to horses [8]. Although TS was ineffective against *Trypanosoma* *cruzi* in some experimental models [20], it successfully eliminated *Trypanosoma* *brucei brucei* parasitemia in sheep, with sustained recovery for up to 100 days post-treatment [21]. Its effect on *T. evansi*, however, remains unexplored.

In Thailand, DA remains the sole approved drug for *T. evansi* treatment. DA acts by inhibiting kinetoplast DNA synthesis [22], but because *T. evansi* can invade the central nervous system (CNS) [23], and DA poorly crosses the blood–brain barrier [22, 24], its effectiveness against CNS infections is limited. Moreover, DA can induce toxicity in horses, and emerging reports of resistance have raised concerns [25]. These factors highlight the urgent need for safer and more effective therapeutic alternatives for controlling equine trypanosomosis [26].

Drug repurposing, the use of existing, well-characterized pharmaceuticals for new therapeutic indications, offers a practical and time-efficient approach to discovering effective anti-trypanosomal agents. Antibiotics such as GMC, CTF, and TS are widely available in Thailand and are frequently prescribed for bacterial infections in horses [27]. However, despite their proven antimicrobial activity and safety in equines, limited or no data exist regarding their efficacy against *T. evansi*, particularly against local Thai strains. Moreover, the previous studies have largely focused on traditional trypanocides using DA, leaving the possible trypanocidal effects of these antibiotics unexplored. Therefore, the *in vitro* evaluation of their inhibitory potential against *T. evansi* represents a critical research gap that could pave the way for novel or adjunct therapeutic strategies in equine trypanosomosis management.

This study aimed to evaluate the *in vitro* inhibitory effects of GMC, CTF, and TS against the Thai strain of *T. evansi* (TEDC 953). The investigation focused on determining the half-maximal effective concentration (EC_50_) of each antibiotic and assessing parasite viability after exposure. By exploring the potential anti-trypanosomal activity of antibiotics that are already widely used and considered safe in horses, this research seeks to identify promising candidates for repurposing or combination therapy with existing trypanocidal drugs such as DA. The findings are expected to contribute to the development of more effective, accessible, and less toxic treatment strategies for equine trypanosomosis in Thailand and other endemic regions.

## MATERIALS AND METHODS

### Ethical approval

The present study was conducted in compliance with the ethical principles for the use of animals in scientific research as outlined by the National Research Council of Thailand and the Faculty of Veterinary Medicine, Kasetsart University, Thailand. All experimental procedures were reviewed and approved by the Institutional Animal Care and Use Committee (IACUC) of Kasetsart University under the protocol titled *“In vitro* evaluation of antibiotic inhibition on *Trypanosoma evansi”* (Approval No. ACKU67-VET-083).

Since this research involved *in vitro* assays using cultured *T. evansi* parasites and did not include the use of live vertebrate animals, no procedures were performed that caused pain, distress, or discomfort to any animal. All biological materials were handled in accordance with the biosafety level 2 (BSL-2) laboratory standards of the Faculty of Veterinary Medicine, Kasetsart University, Bangkok, Thailand.

The study ensured strict adherence to institutional biosafety protocols, proper waste disposal, and the use of personal protective equipment by all personnel. The culture materials and chemical reagents were disposed of following the Kasetsart University Laboratory Biosafety Guidelines.

Ethical oversight by the IACUC guarantees that the research design, execution, and data handling complied with national and international standards. These standards include the Organization for Economic Co-operation and Development good laboratory practice principles, where applicable, in the Animal Research: Reporting *In Vivo* Experiments 2.0 guidelines.

### Study period and location

The experiments were conducted between April 26 and September 12, 2024, in the BSL-2 Laboratory, Faculty of Veterinary Medicine, Kasetsart University, Bangkok, Thailand.

### Parasite strain and *in vitro* cultivation

The Thai strain of *T. evansi* (TEDC 953) was used in this study [28, 29]. Trypomastigotes were cultured in Hirumi’s Modified Iscove’s Medium 9 (HMI-9) medium prepared with Iscove’s Modified Dulbecco’s Medium (IMDM) (IMDM; Gibco, USA, Cat. No. 12440046) supplemented with 0.05 mM bathocuproine disulfonic acid (Sigma-Aldrich, Cat. No. B1125), 1.5 mM L-cysteine (Merck KGaA, Darmstadt, Germany, Cat. No. C7352), 1 mM hypoxanthine (Merck KGaA Cat. No. H9636), 0.16 mM thymidine (Merck KGaACat. No. T1895), 1.4% 2-β-mercaptoethanol (Merck, USA), 1 mM sodium pyruvate (Gibco, Thermo Fisher Scientific, Waltham, MA, USA,), and 1 mM penicillin-streptomycin (Gibco, Thermo Fisher Scientific, Cat. No. 15140–122). The culture was further enriched with 20% horse serum (Gibco, Thermo Fisher Scientific). Parasites were maintained under stationary conditions at 37°C, 5% CO_2_ and 75% humidity for 48 h [28]. Parasite viability was monitored daily using an inverted microscope (Olympus Corp., Japan).

### Antibiotic and trypanocidal drug preparation

The reference trypanocidal drug DA (Berenil R.T.U., USA) was prepared in IMDM at a stock concentration of 7 mg/mL [30]. CTF (Sigma-Aldrich, USA) was dissolved in dimethyl sulfoxide (DMSO) at 200 mg/mL [31], GMC (Sigma-Aldrich) in IMDM at 500 mg/mL [32], and TS (Cotrimoxazole; Sigma-Aldrich) in DMSO at 50 mg/mL [33].

DA and GMC stocks were stored at 4°C under light protection, whereas CTF and TS stocks were stored at −20°C. In all assays, final concentrations of IMDM and DMSO did not exceed 10% and 1%, respectively. Working dilutions were prepared in HMI-9 medium (15 serial tenfold dilutions) to yield concentration ranges as follows:

- DA: 7 to 7 × 10^-14^ mg/mL
- GMC: 50 to 5 × 10^-13^ mg/mL
- CTF: 2 to 2 × 10^-14^ mg/mL
- TS: 5 × 101 to 5 × 10^-15^ mg/mL.

Concentration ranges were selected based on safety limits for equine use and previous experimental data. All solvent choices followed the manufacturer’s recommendations.

### *In vitro* drug-inhibition assay

*T. evansi* trypomastigotes were enumerated using a modified Bürker counting chamber [34], and the inoculum was adjusted to 5 × 10^3^ trypomastigotes/µL [35–37]. *In vitro* inhibition assays were performed in sterile 96-well cell culture plates (Eppendorf, Germany). Each well contained 100 µL of the drug dilution in HMI-9 medium and 5 × 10^3^ trypomastigotes. Plates were incubated at 37°C, 5% CO_2_, and 75% humidity for 48 h.

DA served as the positive control, and untreated parasites served as the negative control. All experiments were conducted in duplicate across three independent trials by a single observer to minimize variability.

### *In vitro* viability assessment

Parasite viability was determined 48 h post-treatment by direct microscopic observation. The number of motile parasites was recorded, and mortality was confirmed by two independent observers.

### Statistical analysis

Parasite counts from the Bürker chamber were used to calculate the percentage inhibition of parasite growth using the following formula:


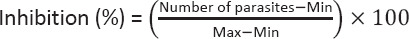


Drug efficacy was analyzed using GraphPad Prism 5 software (GraphPad Software, Inc., San Diego, CA, USA). Nonlinear regression analysis (four-parameter logistic model) was applied to determine the half-maximal effective concentration (EC_50_) for each drug [38]. The regression equation used was:


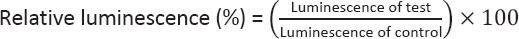


Where Y represents the drug response (% inhibition), Max and Min are the upper and lower asymptotes (100% and 0%), and X is the logarithm of drug concentration.

## RESULTS

### Antibiotic inhibition of *T. evansi* TEDC 953 Thai strain *in vitro*

The *in vitro* growth inhibition assay demonstrated that GMC and CTF effectively suppressed the proliferation of *T. evansi* (Thai strain TEDC 953) after 48 h of incubation. In contrast, TS showed no inhibitory activity against *T. evansi* under the experimental conditions.

The half-maximal effective concentration (EC_50_) values obtained for the tested compounds were 1.25 × 10^-5^ ± 3.90 × 10^-6^ mg/mL for DA, 0.22 ± 0.08 mg/mL for GMC, and 0.08 ± 0.05 mg/mL for CTF (Table 1). These results indicate that both GMC and CTF possess notable *in vitro* trypanocidal activity, although their potency was lower than that of the reference trypanocide DA.

**Table 1:** The EC_50_ values of different medicines against *Trypanosoma evansi* growth *in vitro.*

Drug	EC₅₀ (mg/mL)	SD
DA	1.25 × 10⁻⁵	±3.90 × 10⁻⁶
GMC	0.22	±0.08
CTF	0.08	±0.05
TS	Ineffective	Ineffective

SD = Standard deviation, DA = Diminazene aceturate, GMC = Gentamicin, CTF = Ceftiofur, TS = Trimethoprim–sulfamethoxazole

**Figure 1:**
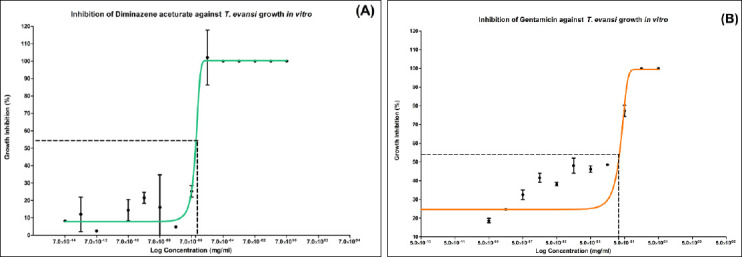
(A) Efficacy of diminazene aceturate in inhibiting *Trypanosoma evansi* growth *in vitro*. (B) Efficacy of gentamicin in inhibiting *T. evansi* growth *in vitro*.

Figure 1 illustrates the concentration-dependent inhibition curves of DA and GMC against *T. evansi* growth. The inhibitory curve for CTF and the EC_50_ determination for TS could not be established due to insufficient inhibitory response during the assay.

### Viability assessment of *T. evansi* after antibiotic exposure

The parasite viability test supported the inhibition results. Complete or near-complete mortality of *T. evansi* trypomastigotes was observed following exposure to DA (7 × 10^-4^ mg/mL), GMC (5 mg/mL), and CTF (0.2 mg/mL) (Table 2). No reduction in parasite viability was detected in the TS-treated or untreated control groups after 48 h of incubation. Overall, these findings confirm that GMC and CTF exhibit measurable *in vitro* trypanocidal activity against the Thai strain of *T. evansi*, whereas TS shows no inhibitory activity under the tested conditions.

**Table 2:** The viability test of antibiotics against *Trypanosoma evansi* Thai strain growth *in vitro.*

Drug	Replicate	D1	D2	D3	D4	D5	D6	D7	D8	D9	D10	D11	D12	D13	D14	D15	D16	D17
DA	Dilutions	7	7×10⁻¹	7×10⁻²	7×10⁻³	7×10⁻⁴	7×10⁻⁵	7×10⁻⁶	7×10⁻⁷	7×10⁻⁸	7×10⁻⁹	7×10⁻¹⁰	7×10⁻¹¹	7×10⁻¹²	7×10⁻¹³	7×10⁻¹⁴	10% IMDM	Control
	R1	−	−	−	−	−	+	+	+	+	+	+	+	+	+	+	+	+
	R2	−	−	−	−	−	+	+	+	+	+	+	+	+	+	+	+	+
GMC	Dilutions	50	5	5×10⁻¹	2×10⁻²	2×10⁻³	2×10⁻⁴	2×10⁻⁵	2×10⁻⁶	2×10⁻⁷	5×10⁻⁸	5×10⁻⁹	5×10⁻¹⁰	5×10⁻¹¹	5×10⁻¹²	5×10⁻¹³	10% IMDM	Control
	R1	−	−	+	+	+	+	+	+	+	+	+	+	+	+	+	+	+
	R2	−	−	+	+	+	+	+	+	+	+	+	+	+	+	+	+	+
CTF	Dilutions	2	2×10⁻¹	2×10⁻²	2×10⁻³	2×10⁻⁴	2×10⁻⁵	2×10⁻⁶	2×10⁻⁷	2×10⁻⁸	2×10⁻⁹	2×10⁻¹⁰	2×10⁻¹¹	2×10⁻¹²	2×10⁻¹³	2×10⁻¹⁴	1% DMSO	Control
	R1	−	−	+	+	+	+	+	+	+	+	+	+	+	+	+	+	+
	R2	−	−	+	+	+	+	+	+	+	+	+	+	+	+	+	+	+
TS	Dilutions	5×10⁻¹	5×10⁻²	5×10⁻³	5×10⁻⁴	5×10⁻⁵	5×10⁻⁶	5×10⁻⁷	5×10⁻⁸	5×10⁻⁹	5×10⁻¹⁰	5×10⁻¹¹	5×10⁻¹²	5×10⁻¹³	5×10⁻¹⁴	5×10⁻¹⁵	1% DMSO	Control
	R1	+	+	+	+	+	+	+	+	+	+	+	+	+	+	+	+	+
	R2	+	+	+	+	+	+	+	+	+	+	+	+	+	+	+	+	+

DA = Diminazene aceturate, GMC = Gentamicin, CTF = Ceftiofur, TS = Trimethoprim–sulfamethoxazole, DMSO = Dimethyl sulfoxide, (−): no inhibitory effect, (+): inhibitory.

## DISCUSSION

### GMC and its trypanocidal potential

GMC, an aminoglycoside antibiotic known for its broad-spectrum antibacterial activity through inhibition of protein synthesis [10], demonstrated a clear trypanocidal effect against the *T. evansi* TEDC 953 Thai strain in the present study. GMC effectively inhibited *T. evansi* proliferation at nanomolar levels, suggesting promising anti-trypanosomal potential. This finding aligns with a previous experimental study in Nubian goats, in which GMC reduced peripheral blood parasitemia for up to 8 weeks post-treatment. Moreover, clinical evidence of successful use of GMC in treating *T. lewisi*-like infections in infants further supports its therapeutic safety and potential as an alternative to conventional trypanocides [12].

The *in vitro* EC_50_ value of GMC against *T. evansi* (0.22 ± 0.08 mg/mL) observed here corresponds well with the recommended safety limits for horses established by the Asia Pacific Center for Animal Health and the National Center for Antimicrobial Stewardship [32]. Previous studies by Bryan [39] and Sirgel *et al*. [40] show that GMC inhibits *Escherichia coli*, *Staphylococcus aureus*, and *Mycoplasma* spp. with EC_50_ values ranging from 0.5 to 16 µg/mL. However, its EC_50_ values for protozoan parasites such as *T. brucei brucei*, *T. evansi*, *Leishmania major* [41], and *Toxoplasma gondii* [42] are typically 100–1,000 times higher than for bacteria, reflecting the greater resilience of protozoa to aminoglycoside action.

Compared with standard trypanocides, GMC displayed lower potency than DA or suramin but comparable or superior efficacy to the other evaluated antibiotics. For instance, chloramphenicol [43] and tetracycline [44] were previously reported to have weak-to-moderate inhibitory effects on *T. evansi* growth (EC_50_ = 50–150 µg/mL), while metronidazole was largely ineffective (EC_50_ > 200 µg/mL) [1]. In contrast, several herbal extracts, such as *Allium sativum* (garlic; 8–20 µg/mL), *Azadirachta indica* (neem; 4–10 µg/mL), *Moringa oleifera* (5–12 µg/mL), and *Nigella sativa* oil (6–10 µg/mL), have demonstrated mild-to-moderate anti-trypanosomal activity [45–48]. These comparisons suggest that while GMC’s trypanocidal activity is moderate, its established safety and pharmacological familiarity make it a feasible candidate for repurposing or combination therapy.

### CTF and comparative inhibitory activity

CTF, a third-generation cephalosporin belonging to the β-lactam antibiotic class [13], also exhibited slight inhibitory effects against *T. evansi* *in vitro*, as confirmed by the viability assays. β-lactam antibiotics act by inhibiting penicillin-binding proteins, thereby interrupting bacterial cell wall synthesis [14]. Although this mechanism does not directly target protozoa, certain β-lactams have been shown to improve trypanocide pharmacokinetics. For example, combining DA with procaine enhanced drug penetration and improved therapeutic outcomes in *T. evansi-*infected buffalo calves [16]. Similarly, the multi-component formulation “Trypan” which combines DA, phenazone, and procaine, has exhibited potent activity against *Trypanosoma congolense*, *Trypanosoma vivax*, *T. brucei brucei*, and *T. evansi* [49].

In this context, the observed partial inhibitory effect of CTF may be attributed to indirect interference with parasite metabolic processes or improved drug bioavailability in combination regimens. These findings warrant further pharmacodynamic studies to elucidate CTF’s mode of action on *T. evansi* and its potential synergism with conventional trypanocides.

### Lack of inhibitory activity of TS

In contrast, TS showed no inhibitory effect on the Thai strain of *T. evansi* under the current experimental conditions. This result is consistent with a previous report by Aregawi *et al*. [1] in mice infected with *T. cruzi*, where TS displayed no therapeutic benefit. The absence of trypanocidal activity can be explained by its mechanism of action: Inhibition of folate synthesis, which is effective against apicomplexan protozoa but not kinetoplastid parasites such as trypanosomes. Nevertheless, combination therapy approaches could enhance TS activity. Earlier studies demonstrated improved efficacy when TS was combined with adjuvant compounds, such as Medicinal Synthetic Aluminum Magnesium Silicate (MSAMS), which stabilizes the drug, prolongs its bioavailability, and modulates drug uptake and metabolic pathways [21, 41]. Such combination strategies merit further exploration for potential synergistic effects against trypanosomosis.

## CONCLUSION

This study demonstrated that GMC and CTF, two antibiotics commonly used in equine medicine, possess notable *in vitro* inhibitory effects against the Thai strain of *T. evansi* (TEDC 953). In contrast, TS showed no measurable inhibition under the tested conditions. The calculated EC_50_ values revealed moderate trypanocidal activity of GMC (0.22 ± 0.08 mg/mL) and CTF (0.08 ± 0.05 mg/mL) when compared with the standard trypanocidal drug DA (1.25 × 10^-5^ ± 3.90 × 10^-6^ mg/mL). Viability assays further confirmed that GMC and CTF induced substantial mortality in *T. evansi* trypomastigotes after 48 h of exposure.

From a practical standpoint, these findings highlight the potential for drug repurposing of GMC and CTF as safe, accessible, and cost-effective therapeutic candidates for managing equine trypanosomosis (Surra) in Thailand and other endemic regions. Given their well-established safety profiles and regulatory approval for veterinary use, these antibiotics could serve as adjuncts to current trypanocidal therapy, helping to mitigate the toxicity and resistance issues associated with DA.

The strength of this study lies in its systematic *in vitro* evaluation of antibiotics not previously characterized for anti-trypanosomal activity against Thai isolates, contributing valuable baseline data for future research. It also provides the first comparative assessment of these drugs’ EC_50_ values against *T. evansi*, establishing a framework for subsequent pharmacological testing.

The present study provides important preliminary insights but also has limitations. The lack of pharmacokinetic data for GMC and the unexplored mechanism of action of CTF against *T. evansi* constrain definitive conclusions regarding their therapeutic potential. While EC_50_ estimation provides valuable quantitative evidence of drug efficacy, *in vivo* pharmacokinetic and pharmacodynamic investigations are essential to confirm clinical applicability.

Future studies should focus on (i) evaluating GMC and CTF in experimental animal models of *T. evansi* infection, (ii) assessing their toxicity and tissue distribution in horses, and (iii) investigating synergistic effects when combined with existing trypanocidal agents such as DA. Such efforts will be critical for developing effective, low-toxicity, and accessible therapeutic options for controlling equine trypanosomosis.

In conclusion, the present study provides promising evidence that GMC and CTF exhibit measurable trypanocidal activity against *T. evansi* *in vitro*. These findings open new avenues for developing safe, repurposed, and combinatory therapeutic strategies to enhance the treatment and control of equine trypanosomosis, thereby improving animal health and reducing economic losses in endemic regions.

## AUTHORS’ CONTRIBUTIONS

KK: Designed and supervised the study and drafted and edited the manuscript. AR and GTN: Contributed equally to this study, performed the experiments, analyzed the data, and drafted and reviewed the manuscript. All authors have read and approved the final version of the manuscript.
